# Suspected involvement of EPTFE membrane in sterile intrathoracic abscess and pericardial empyema in a multi-allergic LVAD recipient: a case report

**DOI:** 10.1186/s13019-015-0305-y

**Published:** 2015-07-17

**Authors:** A. Kornberger, V. Walter, M. Khalil, P. Therapidis, B. Assmus, A. Moritz, A. Beiras-Fernandez, U. A. Stock

**Affiliations:** 1 Department of Thoracic and Cardiovascular Surgery, Johann Wolfgang Goethe University, Theodor-Stern-Kai 7, Frankfurt am Main, Germany; 2 Department of Cardiology, Johann Wolfgang Goethe University, Theodor-Stern-Kai 7, Frankfurt am Main, Germany

**Keywords:** LVAD, ePTFE membrane, sterile abscess, pericardial empyema

## Abstract

Device-related infections in recipients of left ventricular assist devices (LVAD) have been recognized as a major source of morbidity and mortality. They require a high level of diagnostic effort as part of the overall burden resulting from infectious complications in LVAD recipients. We present a multi-allergic patient who was treated for persistent sterile intrathoracic abscess formation and pericardial empyema following minimally invasive LVAD implantation including use of a sheet of e-polytetrafluoroethylene (ePTFE) membrane to restore pericardial integrity. Sterile abscess formation and pericardial empyema recurred after surgical removal until the ePTFE membrane was removed, suggesting that in disposed patients, ePTFE may be related to sterile abscess formation or sterile empyema.

## Background

Device-related infections in LVAD recipients remain major complications associated with relevant morbidity and mortality. In the Mechanical Assistance for the Treatment of Congestive Heart Failure (REMATCH) trial, sepsis was the most common cause of death, accounting for 41 % of the fatal outcomes in the patient group who received LVADs [1]. Evaluation of infection in the framework of the ADVANCE Bridge to Transplant trial showed that driveline exit site infections occurred in 16.9 % and sepsis in 17.2 % of the study population, with fatal outcome in 17.5 % of those who developed sepsis [2].

The HVAD (HeartWare Inc., Massachusetts, USA) is a new generation continuous-flow LVAD that is small enough to fit entirely within the pericardium without need for creation of a pump pocket. When the device is implanted by a minimally invasive approach through a left anterolateral minithoracotomy, lateral closure of the pericardium to cover the device is usually not possible, and e-polytetrafluoroethylene (ePTFE) membrane or bovine pericardium may be used to prevent contact between the device protruding from the opening in the pericardial sac and the lung or the thoracic wall.

We present a case where a sterile intrathoracic abscess and intrapericardial empyema developed following closure of the pericardium using ePTFE membrane in a multi-allergic patient.

## Case presentation

A 43 year old male with a history of multiple allergies including severe lactose intolerance presented with dilated cardiomyopathy (Intermacs level IV) secondary to the cardiotoxicity of polychemotherapy with doxorubicin, cyclophosphamide and vincristine for childhood laryngeal rhabdomyosarcoma. Implantation of an HVAD as a bridge to transplant was performed as an elective minimally invasive off-pump procedure through an L-shaped partial upper sternotomy through the third intercostal space and a left anterolateral minithoracotomy. An ePTFE membrane (Gore® Preclude® Pericardial Membrane, W. L. Gore & Associates, Inc. Flagstaff, USA) was used to restore pericardial integrity. The surgical procedure and the postoperative course were uneventful. Perioperative antimicrobial prophylaxis consisted of cefuroxime and vancomycin for 48 h.

After a hospital stay of 20 days, he was discharged. 3 weeks later, he was readmitted for suspected driveline infection with driveline exit swabs positive for *Staphylococcus epidermidis*. Computed tomography (CT) was performed but showed no signs of deep driveline infection. He was treated with intravenous linezolid (2 × 600 mg/d) and discharged on oral linezolid 10 days later*.* After 13 days, i.e. on post-implant day 65, he was readmitted for VAD-associated infection with significant quantities of spontaneous putrid discharge from the anterolateral thoracotomy wound. At the time of readmission, his clinical condition was not relevantly compromised apart from discomfort and tenderness at the thoracotomy site. LVAD parameters were within normal ranges. Anti-infective therapy was altered to intravenous rifampicin (3 × 300 mg i.v./d) and daptomycin (1 × 500 mg i.v./d), and he was immediately scheduled for surgical exploration.

After opening of the left anterolateral minithoracotomy wound, an accumulation of pus was found in the space between the pericardial sac containing the LVAD and the thoracic wall. The ePTFE membrane loosely covered the HVAD without adhering to the surrounding lung and/or thoracic wall. After removal of the pus and rinsing of the wound, vacuum therapy (VAC®, KCI, Texas, USA) was initiated. One day later, surgical revision was performed for ongoing bleeding from the wound. This time, an accumulation of liquid was palpable under the ePTFE membrane, and after incision of the membrane, a considerable quantity of viscous, putrid discharge drained from within the pericardial sac. An extensive series of swabs obtained during these surgical procedures all returned negative, as did several subsequent swabs taken from the driveline exit and the depth of the wound and a number of blood cultures.

The patient was admitted to the medical intensive care unit (ICU), and his heart transplant (HTX) listing status was upgraded to high urgency for mediastinitis and device infection. He remained hemodynamically stable without requiring inotropic or vasopressor support. After 8 days of vacuum therapy, the wound was therefore rinsed and closed.

**Fig. 1:**
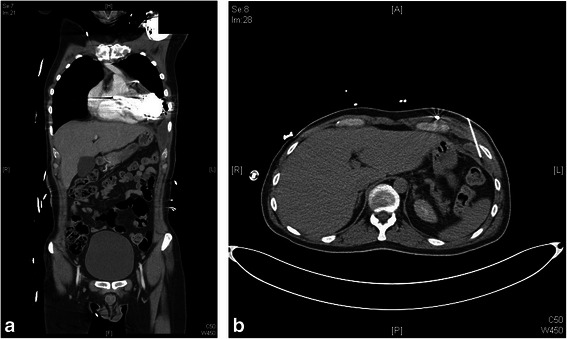
**a** CT showing abscess reaching up to and adjoining the HVAD. **b** CT showing CT-guided abscess drainage

When purulent discharge reoccurred 6 days after wound closure, CT showed an intrathoracic abscess extending up to the HVAD (Fig. 1a). CT-guided drainage of the abscess (Fig. 1b) and of a pleural effusion were performed, and antimicrobial therapy was extended to include piperacillin/tazobactam (3 × 4.5 g/d). 6 days later, repeat CT showed that the abscess had regained its previous size. The patient once again underwent surgery, this time comprising removal not only of the abscess and the intrapericardial empyema, but also of the ePTFE membrane, which had come to be suspected to be implicated in the complication. The foam dressing was changed 4 days later, and the wound was finally closed after 3 further days of vacuum therapy (VAC®, KCI, Texas, USA).

Examination of a multitude of swabs and cultures obtained during all interventional and surgical procedures as well as PCR testing for bacteria and fungi of the ePTFE membrane failed to yield a causative organism. A swab that was taken from the chest drain several days after the initial surgical procedure returned positive for *Pseudomonas aeruginosa* grown on enriched medium. However, *Pseudomonas aeruginosa* was not cultured from any other site, leaving the diagnostic value of this particular swab doubtful. Nevertheless, antimicrobial therapy was altered to include levofloxacin (2 × 500 mg/d) one day after the first wound closure. From two swabs taken from the chest drains two days after the final wound closure, one remained negative while the other yielded Candida spp. sensitive to fluconazole. Contamination was suspected, however, as *Candida albicans* had not been grown from any of the intrathoracic swabs but was present in a large patch of axillary intertrigo that reached down to within a few centimeters from the thoracotomy wound and the chest drain exit site.

**Fig. 2:**
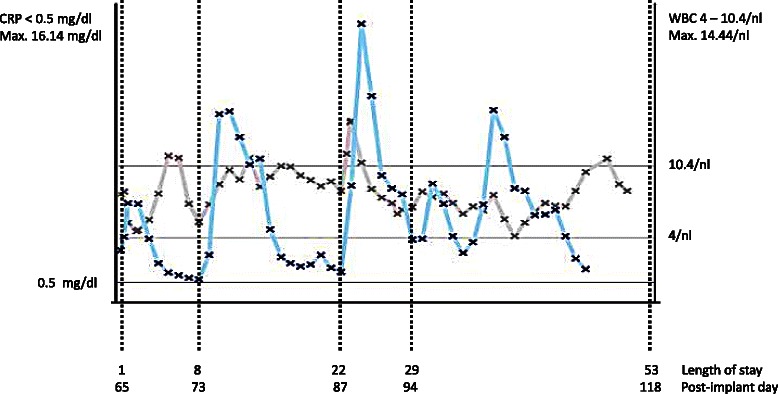
Infection markers (CRP and WBC count) during the 53 day hospital stay. Day 1: surgical exploration and initiation of underpressure therapy; day 8: wound closure; day 22: extensive surgical re-exploration; day 29: wound closure

The anti-infective regimen, which had previously been altered to an empirical combination of rifampicin, cotrimoxazol and ciprofloxacin, was supplemented by fluconazole (1x200 mg/d) when *Candida albicans* was also cultured from the tip of a central venous catheter one week later, accompanied by a final spike in CRP on the same day, even though a blood culture obtained from the same catheter on the same day was negative and wound swabs and driveline exit swabs also kept returning negative. From then on, CRP kept dropping, and the WBC remained within normal ranges (Fig. 2)

A final CT scan showed that abscess formation had not recurred. As the patient’s clinical condition, his surgical wounds, the driveline exit site as well as his infection markers continued to be inconspicuous, his HTX urgency status was downgraded, and he was transferred to the general ward and discharged to self-care on post-implant day 118 after a total hospital stay of 53 days including 46 days in the medical ICU/IMC. His antibiotic regimen at discharge consisted of fluconazole (1 × 200 mg/d), ciprofloxacin (2 × 500 mg/d), sulfomethaxozole/trimethoprim(2 × 960 mg/d) and rifampicin (2 × 450 mg) to be continued for a total of 14 days.

Follow-up CT on post-implant day 135 showed no recurrence of abscess formation. His driveline exit site and thoracotomy wound were inconspicuous. Blood and urine cultures, as well as routine screening swabs and a swab from the driveline exit site returned negative for bacteria and fungi. During his most recent outpatient clinic visit on post-implant day 359, i.e. 265 days after final closure of the anterolateral thoracotomy wound, his clinical condition and infection markers were still inconspicuous.

## Discussion

Management of infections in VAD recipients remains a challenging task, with the infections specific to or requiring particular considerations in VAD recipients ranging from superficial driveline exit site infection to devastating entities such as pump and/or cannula infection, endocarditis and sepsis. The range of treatment options correspondingly covers the entire spectrum from local treatment to major cardiac surgery including device removal with or without temporary mechanical circulatory support, VAD-to-VAD exchange or urgent heart transplantation [3–6].

LVAD exchange for infection was found to be associated with relevant morbidity and mortality, as well as a considerable risk of recurring infection [7–10]. The cases of VAD exchange for infection reported to date, however, mostly concerned the HeartMate II. Our patient, in contrast, received an HVAD, which renders the situation somewhat different in that the pump housing is attached directly to the heart and the entire system is completely contained within the pericardial sac. Regardless of the implantation characteristics, however, the risks of retaining the VAD and those of timely VAD exchange under controlled conditions before development of frank mediastinitis and sepsis need to be carefully weighed against each other. At the same time, the likelihood of availability of a donor organ for any given patient must be considered before proceeding with one or the other approach.

Studies of the microbiology of VAD-associated infection showed the majority of infections to be caused by staphylococcal organisms [11–16]. The share of culture-negative infections reported is low, with inability to identify the causative organism, like in other non-valvular cardiovascular device related infections, being frequently attributed to previous exposure to antibiotics diminishing the sensitivity of subsequent microbiological studies [15, 17].

Upon admission for suspected purulent device infection, antimicrobial treatment in our patient was escalated to a combination of daptomycin and rifampicin in the absence of recent microbiological findings guiding anti-infective therapy. *Staphylococcus epidermidis*, after all, was not cultured from any site after driveline exit site swabs had briefly been positive during the previous hospital stay, and the infectious complication had developed while the patient was on anti-infective treatment with linezolid.

Daptomycin was chosen as it had previously proven effective in the treatment of patients presenting with multi-drug resistant organisms after VAD implantation and for its unique ability to penetrate into biofilms on cardiovascular devices such as LVADS [18–20]. Rifampicin was added for its synergistic effect with daptomycin [21]. Subsequently, anti-infective therapy was altered to different broad combinations of antibiotics that did not, however, comprise antifungals until the last few days of the hospital stay.

In retrospect, a considerable diagnostic effort (Table 1) was dedicated to identifying a causative organism. This is reflected by a total of 18 superficial, deep, intrathoracic and intrapericardial wound swabs including swabs from the LVAD surface subjected to microbiological examination during a hospital stay of 53 days. PCR testing for bacteria and fungi was additionally performed on fluid obtained by CT guided aspiration from the intrathoracic abscess and on a specimen from the ePTFE membrane removed during surgical exploration. Results of PCR testing, all intrathoracic and intrapericardial swabs including swabs from HVAD surface, as well as all swabs taken from the driveline exit site were negative. The only swabs that returned positive were taken by undefined methods and under non-standardized conditions from closed chest drainage systems and must be considered of doubtful diagnostic value.

**Table 1:** Microbiological testing including routine screening for multi-resistant organisms performed during hospital stay of 53 days

Summary of microbiological testing		
Test	No.	Result
Superficial, deep, intrathoracic and intrapericardial wound swabs	18	Negative
Driveline exit site swabs	6	Negative
Swabs from chest drains	3	1 positive for Pseudomonas aeruginosa^a^
		1 positive for Candida spp.^a^
Culture from abscess fluid	1	Negative
Culture from pleural effusion	1	Negative
Blood culture	3	Negative
Microbiological examination of ePTFE membrane	1	Negative
PCR for bacteria and fungi of ePTFE membrane	1	Negative
PCR for bacteria and fungi of fluid aspirated from abscess	1	Negative
PCR for bacteria and fungi of pleural effusion	1	Negative
Nose swabs	6	Negative
Rectal swabs	6	1 positive for Enterobacter cloacae
		1 positive for E. coli
Anal swab	1	Positive for E. coli, Klebsiella pneumonia, Candida albicans and Candida utilis
Stool culture	1	Positive for Candida albicans and Candida non-albicans
Urine culture	7	Negative
Tip of central venous catheter	3	1 positive for Candida albicans
Axillary skin swab	1	Positive for Candida albicans

^a^Suspected contamination

When microbiological studies failed to identify a causative organism, the presence of the ePTFE membrane, in combination with the patient’s severe allergic disposition, came to be suspected of being involved in the complication.

ePTFE is a material widely used in cardiac surgery and appreciated for its chemical inertness and biocompatibility. Whether or not it is superior to a number of alternatives available in preventing adhesions and providing a clear plane of dissection at redo-surgery or, in the case of patients receiving LVADs as bridge to recovery or transplant, at LVAD explantation or heart transplantation is a subject of ongoing debate [22–26]. Permanent presence in the mediastinum of non-resorbable foreign material additionally poses questions with regard to its implications in terms of infection. However, over the past two decades, different studies concluded that ePTFE membrane can safely be used in cardiac surgery to limit adhesions without increasing the risk of infection [27–30].

As infection is a particular issue with VAD recipients, questions were also raised with regard to the material to be chosen for covering the components of the various devices during the implantation procedure. A number of studies were dedicated to the use of ePTFE membrane in the context of VAD implantation and found that it significantly facilitated explantation [31–34].

Leprince et al. [34], studying 23 patients in whom ePTFE membranes had been used to cover LVADs or total artificial hearts, found that infection had occurred in only one case. They concluded that ePTFE membranes can be used to limit adhesion between tissues and device surfaces without increasing the risk of infection. Safety of ePTFE membrane was similarly emphasized by Vitali et al. [33], who used ePTFE pericardial membrane in 20 patients implanted with a Novacor assist device. In contrast, Holman et al. [32] reported membranes in two of seven patients to have become infected with *Staphylococcus aureus* but nevertheless advocated the use of ePTFE membrane.

In our patients, who routinely undergo minimally invasive HVAD implantation, ePTFE membrane or, more recently, bovine pericardium are used to close the lateral gap in the pericardial sac to prevent direct contact between device and lung or thoracic wall rather than to achieve pericardial closure and facilitate re-sternotomy after full median sternotomy. When the anterolateral thoracotomy wound in our patient was reopened on post-implant days 65 and 87, neither adhesion to surrounding structures nor any changes to the membrane itself were observed, but an accumulation of viscous purulent fluid beneath the membrane, in addition to intrathoracic abscess formation, represented a highly alarming finding.

Finally, however, we succeeded neither in identifying a causative organism nor in proving involvement of the ePTFE membrane in the development of the complication with absolute certainty. But given the patient’s severe allergic disposition, the fact that neither pericardial empyema nor intrathoracic abscess formation recurred after removal of the ePTFE membrane may be interpreted as suggestive of involvement of the membrane in the complication.

The level of diagnostic effort undertaken in this particular patient was motivated by a high degree of awareness of the potentially detrimental consequences of VAD infection. Even though the complication resolved without major cardiac surgery being required, the patient had a hospital stay of 53 days most of which he spent at the medical ICU/IMC, thus illustrating that infection in a growing population of VAD recipients represents a veritable burden and relevant economic factor the impact of which grows with the severity of infection and extent of diagnostics and therapeutic interventions required.

## Conclusions

In disposed patients, ePTFE may be related to sterile abscess formation or sterile empyema. This case, together with another case where infection developed in a patient in whom ePTFE membrane was used to cover an HVAD, motivated us to abandon the use of ePTFE membrane. We now use bovine pericardium for restitution of pericardial integrity after minimal invasive HVAD implantation through an anterolateral minithoracotomy.

## Consent

Written informed consent was obtained from the patient for publication of this case report and any accompanying images. A copy of the written consent is available for review by the Editor-in-Chief of this journal.

## Abbreviations

CRP: C-reactive protein
CT: computed tomography
ePTFE: extruded polytetrafluoroethylene
HTX: heart transplantation
HVAD: ventricular assist device of HeartWare Inc.
ICU: intensive care unit
IMC: intermediate care unit
LVAD: left ventricular assist device
PCR: polymerase chain reaction
VAD: ventricular assist device
WBC: white blood cells
